# Saturation of muscle and peripheral oxygen, blood pressure, and perceived exertion in individuals exposed to repetitive muscle work

**DOI:** 10.47626/1679-4435-2026-1521

**Published:** 2026-02-27

**Authors:** Guido Clemente Solari Montenegro, Monserrat Elliet Rivera Iratchet, Juan Ignacio Guerrero Henríquez, Martin Vargas Matamala, Yerko Andrés Villagra Jofré

**Affiliations:** 1 Universidad de Antofagasta, Centro de fisiologia y medicina de altura (FIMEDALT), Antofagasta, Chile; 2 Universidad de Antofagasta, Facultad de Ciencias de la Salud, Antofagasta, Chile

**Keywords:** blood pressure, oxygen saturation, physiology, ergonomics, near-infrared spectroscopy., presión sanguínea, saturación de oxígeno, fisiología, ergonomía, espectroscopía infrarroja corta.

## Abstract

**Introduction:**

Sex-related physiological responses to repetitive load-bearing work are of interest due to the limited available evidence and their relevance to occupational health.

**Objectives:**

To investigate potential sex-related differences in selected physiological and perceptual variables during the performance of a repetitive upper-extremity task.

**Methods:**

A non-probabilistic sample of 20 young adults (10 men and 10 women) was recruited. Participants, in a standing position, performed repetitive work cycles every 2 seconds using their dominant upper limb with the elbow flexed at 90° elbow flexion, while handling loads equivalent to 10%, 20%, and 30% of their maximum voluntary isometric contraction. Local muscle oxygenation was monitored at 0, 10, and 20 minutes in the finger flexors and elbow flexors. Peripheral oxygen saturation, blood pressure, and perceived exertion were also assessed.

**Results:**

No statistically significant differences were observed in muscle oxygen saturation; however, men showed slightly higher values. A significant sex difference in perceived exertion was identified only in the elbow flexors at 10% load at 10 minutes (p = 0.029). Blood pressure values were significantly higher in men, but only at minute 20 with the 10% and 20% loads.

**Conclusions:**

Physiological responses to repetitive work showed minimal sex-related differences. Local and global adaptations were similar, with only minor variations in perceived exertion. Future research should explore sex-related variability in samples representing the average working-age population and using alternative operational models.

## Resumen

**Introducción:**

Las respuestas fisiológicas al trabajo repetido con carga según el sexo son un tema de interés, dada la escasa información disponible y su relevancia en la salud laboral.

**Objetivos:**

Investigar diferencias asociadas al sexo en determinadas variables fisiológicas y perceptivas al ejecutar una tarea repetitiva de la extremidad superior.

**Métodos:**

Se utilizó un muestreo no probabilístico de 20 jóvenes (10 hombres y 10 mujeres) quienes, en posición supina, ejecutaron ciclos de trabajo repetitivo cada 2 segundos con la extremidad superior dominante, con el codo flexionado a 90°, desplazando una carga del 10, 20 y 30% de su contracción isométrica voluntaria máxima. Se monitoreó la oxigenación muscular local a los 0, 10 y 20 minutos en los músculos flexores de los dedos y flexores del codo, así como la saturación periférica de oxígeno, presión arterial y percepción del esfuerzo.

**Resultados:**

No se identificaron diferencias estadísticamente significativas en la saturación muscular de oxígeno; no obstante, los hombres registraron valores levemente superiores. Hubo diferencia estadística en la percepción de esfuerzo únicamente en los flexores del codo al 10% de carga a los 10 minutos (p = 0,029). La presión arterial presentó valores significativamente más altos en los hombres, pero solo a los 20 minutos con carga del 10% y 20%.

**Conclusiones:**

Las respuestas fisiológicas al trabajo repetitivo mostraron mínimas diferencias según el sexo. Las adaptaciones locales y globales fueron similares, con variaciones menores en la percepción del esfuerzo. Se sugiere explorar la variabilidad sexual en muestras con edades de población laboral promedio y mediante modelos operacionales alternativos.

## INTRODUCTION

Musculoskeletal disorders (MSDs) are defined as alterations affecting muscles, tendons, bones, nerves, joints, or ligaments that compromise motor or sensory function. These disorders may be acute (trauma, impacts, or falls), or cumulative, arising from repeated exposure to a movement [1]. When MSDs are caused, exacerbated, or accelerated by occupational exposure, they are referred to as work-related MSDs (WMSDs) [2] and may lead to significant pain and suffering among affected workers, as well as decreased productivity and work quality and potential long-term disability [3].

Models designed to explain the occurrence of these disorders have incorporated causal variables and mechanisms, have examined risk factors and their interrelationships, and have even suggested that certain sociodemographic characteristics may influence the development of WMSDs. In this context, biological sex and muscle oxygenation have been identified as key factors [2]. However, the actual impact of these measures on epidemiological trends and assessment quality has not been sufficiently evaluated and has been criticized [4]. Indeed, in Chile in 2022, WMSDs accounted for 43% of all reported occupational diseases (out of 47,462 cases), despite the implementation of regulatory frameworks aimed at their control [4,5].

Some authors have investigated the responses in muscle oxygen saturation (SmO₂), peripheral oxygen saturation (SpO₂), blood pressure, and perceived exertion among men and women during repetitive tasks involving progressive upper-extremity loading. However, there is still a lack of studies focusing on the simultaneous identification of physiological and perceptual differences between sexes that may contribute to a better understanding of sex-disaggregated WMSDs [6,7].

Furthermore, biological factors may exert a greater influence than sociocultural factors on the development of WMSDs, particularly in the context of cardiovascular regulation, where sex-based physiological differences have been consistently documented [6,8].

Longitudinal studies have shown a faster increase in blood pressure in women compared with men, suggesting the involvement of sex-specific physiological mechanisms. Likewise, sex-related variations in perceived exertion have also been described. Men and women exhibit different perceptual response curves during submaximal exercise, with women reporting greater perceived effort at higher muscle workload [6].

In addition to cardiovascular and perceptual differences, sex-related variability in SmO₂ during physical effort has also been observed. Recent studies indicated that men and women show distinct SmO₂ responses during incremental exercise, with trained men tending to experience more pronounced decreases, whereas women maintain higher SmO₂ levels at equivalent relative workloads (p < 0.001) [9]. Similarly, during intermittent contraction tasks, men tend to exhibit faster reductions in SmO₂ compared with women [10,11].

Overall, the combined behavior of these physiological and perceptual variables - particularly during repetitive and progressively demanding work - remains poorly investigated, especially in occupational environments, where workers may be exposed to involving cyclic movements, submaximal work, and prolonged sustained effort [12,13]. In these settings, elucidating the interaction between locales responses (e.g., SmO₂), systemic indicators (e.g., SpO₂ and blood pressure), and perceived exertion may yield important information regarding fatigue, reduced performance, and development of MSDs. Addressing this knowledge gap has practical implications for occupational health policies, as an integrated physiological approach could improve risk assessment and guide targeted prevention strategies.

Moreover, the identification of sex-specific response patterns may help support tailored interventions, workload adjustments, and return-to-work policies, thereby reducing the incidence of WMSDs. Accordingly, the study hypothesizes that during repetitive muscle work performed at 10, 20, and 30% of maximum voluntary isometric contraction (MVIC), men show slightly higher SmO₂ and blood pressure values, along with higher perceived exertion, compared with women.

The aim of this study was to identify physiological and perceptual responses - specifically SmO₂, SpO₂, blood pressure, and perceived exertion - in men and women performing a repetitive, dynamic, upper-extremity task with progressively increasing load.

## METHODS

### DESIGN

A quasi-experimental, quantitative, correlational design was employed to investigate potential significant differences in dependent variables (SmO₂, blood pressure, and perceived exertion) and independent variables (10%, 20%, 30% of MVIC) between men and women performing a protocol-based repetitive task. Experimental conditions were standardized with respect to work duration, repetition frequency, rest intervals between sessions, and technical actions or movement patterns during the work cycle, with the task executed at 90° elbow flexion in a sustained standing position.

### PARTICIPANTS

Twenty participants were recruited and equality distributed into two groups (10 women and 10 men). Eligibility criteria included age between 18 and 25 years, provision of informed consent, and absence of functional or musculoskeletal impairments identified during preliminary screening. Upper-extremity function and disability were assessed using the shortened Disabilities of the Arm, Shoulder and Hand (QuickDASH) questionnaire [14], and the presence and distribution of musculoskeletal symptoms across different body regions using the Nordic Musculoskeletal Questionnaire.

Exclusion criteria comprised the presence of pain, any MDS within the 30 days prior to protocol implementation, medically uncontrolled arterial hypertension, body mass index ≥ 35 kg/m^2^, concurrent participation in work or sports activities involving upper-extremity tasks, and a high risk of absenteeism from study sessions.

### ETHICS

The study protocol, associated procedures, and informed consent were reviewed and approved by the Scientific Research Ethics Committee of Universidad de Antofagasta (approval certificate No. 493/2024).

### INSTRUMENTS

MVIC of the dominant upper extremity was assessed at baseline for finger flexors (grip strength) and elbow flexors. Grip strength was measured in a seated position with the elbow flexed to 90° and the forearm in a neutral supinated position, using a calibrated Baseline^®^ LiTE^®^ hydraulic dynamometer (200 lb capacity) equipped with an analog display and peak-hold indicator. Three maximum voluntary contractions were performed, each sustained for 3 seconds, with a 60-second rest interval between trials. The indicator was reset to zero before each attempt. The final value considered was the highest value obtained across the three trials.

Elbow flexor strength was assessed with participants seated, their back fully supported against the wall, shoulder positioned at 0° abduction, elbow flexed at 90°, and forearm supinated, while holding the handle of the digital dynamometer with the dominant hand. A Tindeq Progressor 200 device, designed for isometric force measurement, was used. Two maximum voluntary contractions were performed, each sustained for 3 seconds, with a 3-minute rest interval between trials to minimize fatigue and ensure full recovery. The final value was the highest value recorded in either trial.

After determining the MVIC for each participant, loads corresponding to 10%, 20%, and 30% were calculated to establish the exact weight of each object (or bag) to be displaced. Throughout all MVIC assessments, particular attention was paid to the standardization of verbal encouragement to ensure sustained maximum effort, consistent body positioning, appropriate examiner handling, and resetting the Baseline^®^ dynamometer to the zero position after each measurement, in accordance with the manufacturer’s technical guidelines.

### PROTOCOL

#### Work surface height

Participants performed the task in a standing position, with the manual activity executed on an electrically height-adjustable table, calibrated according to each participant’s anthropometric measurements (anterior superior iliac spine to floor), ensuring an elbow joint angle of 90° (± 5°) during task execution.

#### Load and object handled during the repetitive task

The manual task involved using a fabric bag with soft handles to allow proper grip coupling. To prevent displacement and excessive volume, the bag was loaded with geometric lead pieces until the target load corresponding to the prescribed percentage of MVIC was reached.

Each participant completed three daily work sessions of 20 minutes each, separated by 1 hour of rest to minimize residual discomfort. In each session, participants performed a cycle task that consisted of grasping the bag handles, lifting the bag 15 cm, and then placing it 15 cm to the left, followed by the reverse movement to complete the cycle. The task was performed at a pace of one cycle every 2 seconds, paced by a metronome programmed for this purpose and continuously supervised by a researcher.

#### Session distribution

During each daily session, the cycle and pace of the manual work remained constant. The only modified variable was the load of the bag being handled, following this sequence: 10% of MVIC in the first session, followed by a 1-hour rest; 20% of MVIC in the second session, followed by a 1-hour rest; and, finally, 30% of MVIC in the third session.

#### Monitoring during sessions

In each session, elbow and finger flexor muscles were simultaneously monitored. Measurements were taken immediately before session onset (minute 0), at minute 10, and at minute 20, immediately upon session completion.

SpO2 was monitored using a pulse oximeter (Nonin 8500 M, Nonin Medical Inc., Plymouth, MN, USA), placed on the index finger. Systolic blood pressure (SBP) and diastolic blood pressure (DBP) were obtained using an ambulatory blood pressure monitor Holter device (Contec ABPM-50) positioned on the upper non-dominant arm. Perceived exertion was evaluated using the Borg Category-Ratio Scale for Rating of Perceived Exertion (Borg CR-10). SmO2 was continuously assessed using non-invasive near-infrared spectroscopy (MOXI monitor), with sensors affixed to the muscle bellies according to the methodology described by Kauppi et al. [15].

### STATISTICAL ANALYSIS

Statistical analysis was performed using SPSS software (version 23.0, IBM Corp., Armonk, NY, USA). Data were compiled into an Excel matrix for descriptive statistical analysis. For all statistical tests, the level of significance was set at α = 0.05. Results are presented as means, SDs, medians, and other relevant descriptive statistics. Normality assumptions were assessed using the Shapiro-Wilk test, and between-sex comparisons of physiological and perceptual responses at different time points and load levels were performed using Mann-Whitney U test and Student’s t tests; Z statistics were applied in selected comparisons.

## RESULTS

Complete datasets were obtained for the elbow flexor muscles across all sessions and load levels. Conversely, for the finger flexor muscles, only data from 10% of MVIC are reported, since sessions at 20% and 30% of MVIC resulted in incomplete recordings owing to participant dropout caused by muscle discomfort during task performance.

As shown in Table 1, baseline (time 0) SmO₂ and SpO₂ distributions during repetitive finger flexor work were similar between men and women. At 10 minutes, perceived exertion (Borg CR-10 score) showed greater dispersion among women, with mean (SD) of 3.14 (8.40) points, compared with men, who showed a mean (SD) of 3.00 (1.90) points. SBP also exhibited high variability in women. Except for perceived exertion (Borg CR-10 score), normality assumptions were violated for the other biological variables; therefore, variance and SD should be interpreted with caution using the Shapiro-Wilk test.

**Table 1:** Distribution of SmO₂, SpO₂, Borg CR-10 scores, SBP, and DBP during repetitive finger flexor work at 10% of MVIC, by time and sex

Biological indicator of exposure	Time (min)	Sex	Mean	Median	Variance	SD
SmO₂ (%)	0	F	92.2	94	31.1	5.6
		M	93.0	95	23.6	4.9
	10	F	89.9	93.5	13.5	3.7
		M	87.2	93.0	116.2	10.8
	20	F	90.8	94.0	155.9	12.5
		M	87.6	93.0	130.6	11.4
SpO₂ (%)	0	F	99.3	99	0.6	0.8
		M	99.4	99	0.2	0.5
	10	F	99.0	99	0.6	0.8
		M	98.8	99.0	0.5	0.6
	20	F	99.2	99	0.2	0.4
		M	98.4	99.0	4.2	2.0
Borg CR-10 score (points)	0	F	1.57	1.0	6.28	2.5
		M	2.57	3.0	2.28	1.5
	10	F	3.14	3.0	71.0	8.4
		M	3.00	3.0	3.66	1.9
	20	F	3.86	4.0	6.40	2.5
		M	3.00	3.0	4.33	2.1
SBP (mm Hg)	0	F	119.7	116.0	70.0	8.4
		M	128.0	127.0	136.8	11.7
	10	F	126.4	121.0	277.6	16.7
		M	129.5	128.0	33.9	5.8
	20	F	122.0	122.0	128.6	11.3
		M	131.7	130.0	117.9	10.8
DBP (mm Hg)	0	F	85.2	87.0	147.2	12.1
		M	87.1	82.0	214.4	14.6
	10	F	82.0	83.0	116.6	10.8
		M	82.1	81.0	64.0	8.0
	20	F	84.7	90.0	81.9	9.1
		M	78.5	75.0	103.6	10.2
Basal metabolic rate	0	F	1,371	1,365	16.3	127.7
Fat mass (%)	0	F	21.93	16.77	31.1	5.6
		M	18.06	18.50	7.9	2.8

F = female; M = male; MVIC = maximum voluntary isometric contraction; SmO₂ = muscle oxygen saturation; SpO₂ = peripheral oxygen saturation.

Mean (SD) SmO₂ during repetitive elbow flexor work was 90% (5%) in men and approximately 77% (28%) in women, with the latter exhibiting greater dispersion. Mean (SD) SpO₂, in turn, remained stable at around 99% (≤ 1%), and perceived exertion at the end of the task (20 minutes) was higher in men, with a mean (SD) Borg CR-10 score of 3.8 (2.4) points at 10% of MVIC, compared with women, whose mean (SD) score was 1.9 (1.9) point, as shown in Table 2. Regarding blood pressure, SBP ranged from 119 0 minute to 132 mm Hg minute 20, showing greater variability among women (SD ≈ 29 mm Hg), whereas DBP remained within a narrower range (≈79-84 mm Hg) (Table 2).

**Table 2:** Distribution of SmO₂, SpO₂, Borg CR-10 scores, SBP, and DBP during repetitive elbow flexor work at 10%, 20%, and 30% of MVIC according to time and sex

Indicator	Time (min)	Sex	Mean	Median	Variance	SD	Mean	Median	Variance	SD	Mean	Median	Variance	SD
			10% of MVIC				20% of MVIC				30% of MVIC			
SmO₂ (%)	0	F	70.3	76	788.0	28.0	90.5	92	92.0	9.6	88.4	89	96.3	9.8
		M	86.9	86.5	73.6	8.5	90.2	90	15.2	3.9	94.0	94	2.0	1.4
	10	F	71.2	83	807.2	28.4	89.4	91	91.0	5.3	87.0	90	104.0	10.1
		M	86.1	86.5	80.9	8.9	87.0	90	61.6	7.8	92.5	92.5	0.5	0.7
	20	F	78.8	79.5	827.0	28.7	87.5	94	94.0	19.5	87.8	95	112.7	10.6
		M	85.5	88.5	93.9	9.6	87.7	91	71.2	8.4	93.0	93	2.0	1.4
SpO₂ (%)	0	F	99.1	99	0.7	0.8	99.1	99	0.3	0.5	99.2	99	0.2	0.4
		M	99.5	100	0.5	0.7	99.7	100	0.2	0.4	99.5	99.5	0.5	0.7
	10	F	99.0	99	0.6	0.8	98.5	98	1.0	1.0	98.6	98	0.8	0.8
		M	99.3	99.5	0.6	0.8	99.2	100	0.9	0.9	98.5	98.5	4.5	2.1
	20	F	99.0	99	0.4	0.6	99.1	99	0.1	0.3	98.6	99	1.3	1.1
		M	99.1	99	0.3	0.5	99.5	100	0.2	0.5	115.4	116	76.3	8.7
Borg CR-10 score (points)	0	F	0.2	0	0.4	0.6	0.55	0	0.7	0.8	1.20	2.0	1.2	1.0
		M	0.2	0	4.0	0.6	0.90	0.5	1.0	1.0	1.00	1.0	2.0	1.4
	10	F	1.1	0	2.5	1.5	2.30	2	3.2	1.8	5.20	5.0	5.2	2.2
		M	2.6	2.5	6.2	2.5	2.20	2	1.8	1.3	2.50	2.5	4.5	2.1
	20	F	1.9	0.5	3.6	1.9	2.10	2	3.36	1.8	4.00	4.0	15.5	3.9
		M	3.8	5.0	5.7	2.4	2.60	3	3.2	1.7	3.00	3.0	8.0	2.8
SBP (mm Hg)	0	F	123.1	125	90.5	9.5	115.4	113	113.0	9.8	115.4	116.0	76.0	8.7
		M	128.2	132.5	159.7	12.6	131.8	124	415.4	20.4	131.0	131.0	2.0	1.4
	10	F	129.2	129	105.2	10.3	124.8	125	91.6	9.6	139.8	138.0	859.0	29.3
		M	128.8	135.0	180.6	13.4	130.4	131	119.2	10.9	118.0	118.0	288.0	16.97
	20	F	118.9	118.5	145.2	12.0	116.7	116	28.1	5.3	124.2	123.0	63.2	7.9
		M	131.8	135.0	157.7	12.6	128.1	129	115.8	10.8	125.0	125.0	2.0	1.4
DBP (mm Hg)	0	F	79.2	82	60.4	7.7	81.7	81	83.0	9.1	78.2	82.0	120.0	10.9
		M	79.0	79.0	72.8	8.5	79.7	80	71.9	8.5	84.0	84.0	50.0	7.0
	10	F	82.9	82.5	49.8	7.1	86.2	89	122.4	11.1	81.4	81.0	137.0	11.7
		M	84.0	81.0	57.3	7.6	84.1	80	256.8	16.0	80.5	80.5	4.5	2.12
	20	F	82.7	82.7	100.0	10.0	80.8	81	107.0	10.3	81.2	82.0	57.2	7.5
		M	78.7	78.0	70.0	8.4	81.2	78	67.5	8.2	85.5	85.5	180.0	13.4

DBP = diastolic blood pressure; F = female; M = male; MVIC = maximum voluntary isometric contraction; SBP = systolic blood pressure; SmO₂ = muscle oxygen saturation; SpO₂ = peripheral oxygen saturation.

Analysis of perceived exertion according to muscle group and load intensity (percentage of MVIC) revealed no sex-related differences for finger flexors at 10% of MVIC at either 10 minutes (t = 0.74; p = 0.451; d = 0.52) or 20 minutes (t = 1.50; p = .271; d = 1.06). Conversely, elbow flexors at 10% of MVIC demonstrated a significant between-sex difference at 10 minutes (t = -1.60; p = 0.029; d = 0.71), which was not maintained at 20 minutes (t = -1.96; p = 0.271; Cohen’s d = 0.88). No significant differences were detected at a load of 20% of MVIC at any time point (d ≤ 0.13) (Table 3).

**Table 3:** Comparison of Berg CR-10 scores between men and women at 10 and 20 minutes during repetitive finger flexor and elbow work at 10%, 20%, and 30% of MVIC

Muscle (%MVIC)	Time (min)	Men Mean (SD)	Women Mean (SD)	t (df)	p-value	d
Finger flexors (10%)	10	2.6 (‑)	3.4 (‑)	t (14) = 0.74	0.451	0.52
Finger flexors (10%)	20	3.0 (‑)	3.86 (‑)	t (11) = 1.50	0.271	1.06
Elbow flexors (10%)	10	2.6 (‑)	1.1 (‑)	t (18) = -1.60	0.029	0.71
Elbow flexors (10%)	20	3.8 (‑)	1.9 (‑)	t (18) = -1.96	0.271	0.88
Elbow flexors (20%)	10	2.2 (1.1)	2.1 (1.5)	t (15) = -0.14	0.264	0.07
Elbow flexors (20%)	20	2.6 (1.3)	2.4 (1.4)	t (15) = -0.26	0.910	0.13
Elbow flexors (30%)	20	3.0 (1.3)	4.0 (1.4)	t = 0.40	0.702	-0.27

d = Cohen’s effect size; MVIC = maximum voluntary isometric contraction; *t* (df) = t statistic with degrees of freedom.

Regarding oxygenation and blood pressure measures, no significant differences in SmO₂, SpO₂, or SBP were observed at 10% of MVIC (Z ranging from -1.70 to -0.30; p ≥ 0.280; r ≤ 0.40). In contrast. DBP showed a significant variation at 20 minutes (Z = -2.10; p = 0.031; r = 0.50), corresponding to a large size effect. The same response pattern was found at 20% of MVIC, with statistical significance detected only DBP at 20 minutes (Z = -2.50; p = 0.010; r = 0.63), highlighting the sensitivity of this parameter to sustained muscle fatigue (Table 4).

**Table 4:** Comparison of SmO₂, SpO₂, SBP, and DBP between men and women at three time points during repetitive elbow flexor work at 10%, 20%, and 30% of MVIC (Mann-Whitney U test)

Percentage of MVIC	Indicator	Time (min)	Z	p-value	r
10%	SmO₂ (%)	0	-1.70	0.080	0.40
	SmO₂ (%)	20	-0.90	0.360	0.21
	SpO₂ (%)	0	-1.07	0.280	0.25
	SpO₂(%)	20	-0.30	0.700	0.07
	SBP (mm Hg)	10	-0.38	0.970	0.09
	DBP (mm Hg)	20	-2.10	0.031	0.50
20%	SmO₂ (%)	0	-0.63	0.528	0.16
	SmO₂ (%)	20	-0.58	0.564	0.15
	SpO₂ (%)	0	-1.67	0.095	0.43
	SpO₂ (%)	20	-1.83	0.067	0.47
	SBP (mm Hg)	10	-0.71	0.479	0.18
	DBP (mm Hg)	10	-1.00	0.317	0.26
	SBP (mm Hg)	20	-0.67	0.504	0.17
	DBP (mm Hg)	20	-2.50	0.010	0.63
30%	SmO₂ (%)	0	-1.00	0.290	-
	SmO₂ (%)	20	-1.00	0.290	-
	SpO₂ (%)	0	-0.30	0.760	-
	SpO₂ (%)	20	0.00	1.000	-
	SBP (mm Hg)	10	-1.00	0.300	-
	DBP (mm Hg)	10	-1.00	0.290	-
	SBP (mm Hg)	20	-0.79	0.420	-
	DBP (mm Hg)	20	-0.15	0.880	-

DBP = diastolic blood pressure; MVIC = maximum voluntary isometric contraction; r = effect size; SBP = systolic blood pressure; Z = Z statistic of the Wilcoxon test.

Sex-based comparison of SmO₂, SpO₂, SBP, and DBP under equivalent conditions revealed no significant differences in SmO₂, SpO₂, or SBP (all p > 0.05). However, SBP measured at 10 minutes during finger flexor work at 10% of MVIC demonstrated a near-significant trend (Z = -1.93; p = 0.054; r = 0.46), whereas DBP at 20 minutes was significantly lower in women (Z = -1.22; p = 0.022), indicating a higher vascular load in men under sustained fatigue (Table 5).

**Table 5:** Comparison of SmO₂, SpO₂, SBP, and DPB between men and women at 0 and 20 minutes of repetitive finger flexor work at 10% of maximum voluntary isometric contraction (Mann-Whitney U test)

Biological indicator of exposure	Time (min)	Z	p-value	r
SmO₂ (%)	0	-0.15	0.800	0.04
SmO₂ (%)	20	-0.90	0.300	0.21
SpO₂ (%)	0	-0.50	0.500	0.12
SpO₂ (%)	20	-0.90	0.300	0.21
SBP (mm Hg)	10	-1.93	0.054	0.46
DBP (mm Hg)	20	-0.29	0.773	0.07
SBP (mm Hg)	0	-1.68	0.093	0.39
DBP (mm Hg)	20	-1.22	0.223	0.29

DBP = diastolic blood pressure; ; r^1^ = tamaño del efecto; SBP = systolic blood pressure; SmO₂ = muscle oxygen saturation; SpO₂ = peripheral oxygen saturation; Z = Z statistic of the Wilcoxon test.

## DISCUSSION

Even in tasks that require lowto moderate-intensity isometric contractions, blood perfusion alone may be insufficient to satisfy metabolic requirement, leading to tissue deoxygenation [16]. Furthermore, maximum muscle contraction is not required to elicit this response, as deoxygenation may occur at contraction at intensities of 30% of MVIC [17]. Consequently, repetitive work with moderate load may contribute to the onset of WMSDs by triggering microvascular dysfunction, favoring anaerobic metabolism, metabolite accumulation (e.g., lactate), and inflammatory processes that ultimately promote structure tissue damage, pain, fatigue, and chronic progression of WMSDs [18].

In the present study, both men and women were unable to tolerate the repetitive finger flexor work at load intensities of 20% and 30% of MVIC, and nearly all participants withdrew prematurely because of muscle discomfort. Completion of the full work period was only achieved under low-load conditions (10% of MVIC) by both sexes. In contrast, all participants successfully completed the repetitive elbow flexor work at all evaluated load levels (10%, 20%, and 30%).

Although no significant sex-related differences were observed across time points and load percentages, SmO₂ was consistently higher in men (mean ≈ 90.0%, SD ≈ 5.0%) than in women (mean ≈ 76.8%, SD ≈ 28.4%), a trend that has been previously reported by other authors when higher work intensities are applied (60% of MVIC) [6]. Likewise, studies examining SmO₂ response in men and women during repetitive handgrip work at 20% of MVIC have indicated greater desaturation in men compared with women within forearm muscle groups. Other investigations [6] examining forearm muscle SmO₂ responses have concluded that the degree of desaturation is influenced both by the load or percentage of MVIC and sex-related differences attributable to muscle mass and morphology. Similarly, several studies have demonstrated that men desaturate more rapidly than women, a phenomenon attributed to variations in muscle fiber composition and mitochondrial properties [19].

Most SmO₂ studies reported in the literature have been conducted in sports and physical activity contexts, and available evidence on muscle oxygenation dynamics suggest that sex-related differences depend primarily on task characteristics such as intensity, duration, repetitiveness, making sex-specific SmO₂ responses difficult to predict. Therefore, additional research is needed in the area, incorporating, among other factors, control of covariates such as muscle perfusion and muscle mass, to clarify the mechanisms underlying SmO₂ responses during repetitive work [20-22].

Regarding perceived exertion, assessed using the Borg CR-10, significant sex-related differences were observed exclusively in the elbow flexor work at 10% of MVIC, in which men reported higher levels of perceived exertion compared with women.

Despite the absence of statistically significant differences, analysis of the full dataset indicates that women consistently tended to report higher perceived exertion than men, especially at lower load levels. Consistent with findings from previous studies, differences in perceived exertion do not necessarily manifest during task execution but become more apparent near the onset of fatigue. The observations suggest that women may rely on distinct neuromuscular strategies to regulate perceived exertion.

The lack of significant differences under the conditions of our study is also consistent with prior literature indicating that sex alone does not constitute a determining factor in perceived fatigue. Instead, it may be influenced by multiple concurrent factors, such as upper-extremity anthropometry, motor coordination, and neuromuscular control strategies [23,24].

With regard to blood pressure, sex-related differences were observed in DBP during repetitive elbow flexor work protocol at 20 minutes with 10% of MVIC) and in SBP during the same protocol at 20 minutes with 20% of MVIC. In all other comparisons, despite the absence of statistical significance, DBP was consistently lower in women, and SBP was higher in men across all protocols.

Consistent with previous research, these results indicate sex-related differences in sensitivity to muscle fatigue, especially during sustained low-intensity tasks. Under these conditions, men tend to demonstrate greater pressor response to isometric exercise, possibly associated with greater activated muscle mass and/or more pronounced sympathetic responses.

Lower DBP in women has also been reported in previous studies that have identified reduced peripheral vascular resistance in women during and after isometric exercise, a finding partially attributable to differences in autonomic control and estrogen-mediated vasodilatation. Such physiological differences may explain the lower hemodynamic load observed in women during prolonged tasks, even at low relative exercise intensities [25].

Furthermore, our findings may be related to the cumulative effect of sustained contraction on muscle blood flow, whereby men exhibit a greater pressor response as a compensatory mechanism to prolonged vascular compression, as previously described in studies involving sustained isometric exercise [26].

In terms of blood pressure, our results showed that DBP was lower in men and SBP was higher in men, with statistically significant differences at specific time points of the repetitive elbow flexor work. This pattern aligns with recent evidence suggesting sex-specific sensitivity in the pressor response during isometric exercise. Specifically, men tend to demonstrate higher pressor responses than women during lowand moderate-intensity static muscle contractions. However, these differences tend to decrease as contraction force increases, indicating that greater active muscle mass and higher absolute strength in men may contribute to enhanced activation of the pressor reflex during sustained tasks [27].

Conversely, lower DBP in women compared with men may be associated with specific vascular mechanisms, as reported by previous studies that have found a reduced pressor response in women during and after isometric exercise. This response has been linked to lower peripheral vascular resistance, partially attributable to enhanced estrogen-mediated vasodilatation and differences in autonomic control. This hemodynamic profile may explain why women experience a lower hemodynamic load, even in the presence of fatigue [28].

The effects of repetitive muscle work on SBP and DBP seem to be time-dependent, such that prolonged muscle contractions, vascular compression, and metabolite accumulation stimulate increased blood pressure. Several studies have reported sex differences in SBP and DBP responses, attributing these differences to vascular and autonomic regulatory mechanisms following isometric exercise. Additionally, some authors have suggested that sustained pressor response in men may represent a compensatory response to prolonged compression of the muscle vascular bed during contraction, which is consistent with findings of more pronounced differences at approximately 20 minutes of work [29].

Lastly, the protocols applied in this study were specifically designed to assess the responses of biological indicators in a small sample using a quasi-experimental design. The findings reported herein do not provide sufficient epidemiological evidence to support preventive strategies or to define occupational health priorities. However, they constitute a relevant basis for future investigations, particularly studies aimed at exploring similar responses employing alternative protocols or those simulating real work models (e.g., loads, rest intervals, and work organization), or investigations conducted in real workplaces aimed at identifying occupational risk factors and contributing to the management of WRMSDs incidence.

## CONCLUSIONS

The results of the present study indicate minimal differences in the physiological responses evaluated between men and women while performing repetitive tasks with variable loads.

Despite the absence of statistically significant differences in SmO₂, higher values were observed in men. Sex differences in perceived exertion emerged only at specific time points, suggesting distinct motor control strategies. In contrast, the most pronounced differences were evident in the hemodynamic response, with men showing significantly higher SBP and DBP values in men at 20 minutes during sustained tasks performed at 10% and 20% of MVIC, respectively. These findings underscore the importance of considering biological sex as a modulating variable in the design of workload protocols and fatigue assessment in occupational and clinical settings.
